# Risk perception and behavior in Egyptian adolescent pesticide applicators: an intervention study

**DOI:** 10.1186/s12889-020-08801-7

**Published:** 2020-05-13

**Authors:** Diane S. Rohlman, Jonathan W. Davis, Ahmed Ismail, Gaafar M. Abdel Rasoul, Olfat Hendy, James R. Olson, Matthew R. Bonner

**Affiliations:** 1 Occupational and Environmental Health, University of Iowa, 145 N. Riverside Drive, Iowa City, IA 52242 USA; 2 Oregon Health and Science University, Portland, OR USA; 3 Faculty of Medicine, Menoufia University, Shebin El-Kom, Egypt; 4 National Liver Institute, Menoufia University, Shebin El-Kom, Egypt; 5 Department of Epidemiology and Environmental Health, State University of New York Buffalo, Buffalo, NY USA; 6 Department of Pharmacology and Toxicology, State University of New York, Buffalo, NY USA

**Keywords:** Pesticides, Behavior change, Educational intervention, Adolescent workers

## Abstract

**Background:**

Adolescents are engaged in agricultural work, including pesticide application, around the world. Adolescent pesticide applicators are more likely to be exposed to pesticides than their adult counterparts because of their application practice and hygiene habits surrounding pesticide use. There is a need for low-cost interventions to reduce pesticide exposure. We evaluated a theoretically-based educational intervention to change perceptions about the risk of pesticide use and hygiene habits during and after pesticide application for adolescent and young adult pesticide applicators in Egypt.

**Methods:**

Young adult and adolescent male pesticide applicators were given a one-hour educational intervention to inform them about the risk of pesticide use and how to reduce pesticide exposure. The median age of participants was 18 years old. Changes in perceived susceptibility and effectiveness were measured with a survey pre and post-intervention (*n* = 119) on the same day. The same survey (*n* = 95) was given 8-months post-intervention to identify sustained effects. Observational checklists of pesticide application practice were also completed during application seasons before and after the intervention.

**Results:**

There was an increase in the proportion of individuals who viewed pesticides as being a long-term health risk (74.7% pre-intervention to 97.9% post-intervention, McNemar test *p* < 0.001). This change remained significant when surveyed at the 8-month follow-up (90.5%, *p* < 0.001). There was also a sustained improvement regarding participants’ views of proper hygiene practice surrounding pesticide application. Applicators were observed wearing goggles, shoes, and masks more frequently post-intervention.

**Conclusion:**

This theoretically-based intervention is an example of a low-cost solution that can improve adolescents’ and young adults’ practices regarding pesticide application and personal hygiene practices during and after pesticide application. The intervention can be applied in other countries with similar safety culture surrounding pesticide application.

## Background

Many children and adolescents throughout the world are engaged in agricultural work, either for pay or on family farms. Studies examining exposure among children have primarily focused on non-occupational or para-occupational factors (e.g.*,* residential use, diet, or take-home exposure) [1–3]. Occupational activities such as mixing, applying, and maintaining equipment, the frequency and duration of spraying, and the use of personal protective equipment (PPE) will impact exposure to pesticides [4–6]. Additionally, personal hygiene factors like bathing or laundering clothing are associated with exposure [7, 8]. Adolescents applying pesticides are more likely to engage in behaviors that can increase their exposure, such as wearing shorts instead of pants, wearing clothing containing pesticide residues, and washing less frequently when compared to adults [9].

In addition to the increased potential for exposure, physiologically a child or adolescent is more susceptible to adverse health effects of pesticides than their adult counterparts [10]. Among adolescent pesticide applicators, exposure to pesticides is associated with increased health symptoms [11, 12], reduced lung function [13], neurobehavioral deficits [14–16], and Attention-Deficit/Hyperactivity Disorder (ADHD) symptoms [17]. Because of the increased likelihood of exposure and risk for adverse health effects, specific interventions targeting adolescent pesticide applicators are warranted.

In this study, we developed an intervention targeted at changing workplace behaviors and hygiene practices that are associated with increased pesticide exposure for adolescent pesticide applicators. Additionally, the intervention educated participants about the health threat of pesticide use and the efficacy of various protective measures to reduce exposure (e.g., avoiding contact with pesticide while mixing and applying, hygiene practices, use of personal protective equipment). Changes in perceptions of pesticide use and measures taken to reduce exposure were evaluated before and after the intervention.

## Methods

### Study population and setting

This study is a pre-post intervention study nested in a longitudinal study of adolescent and young adult pesticide applicators in Egypt. The original longitudinal study started in 2014 and continued to 2017, with follow-up testing in 2018 and 2019. Adolescents under the age of 19 who worked for the Ministry of Agriculture to spray pesticides on cotton in the Nile delta were recruited in 2014 and 2015 from four field stations (Quesna, Shohada, Tala, and Berket El-Sabe’) in Menoufia Governorate, Egypt. A small subset of applicators had been enrolled in a previous longitudinal study (*n* = 13, 8.4%) and were invited to participate in the current study [14]. Results were replicated excluding these individuals and no difference in the results were found. Applicators had the following job responsibilities surrounding pesticide use: mixing pesticides, filling backpack sprayers, application of pesticides, and cleanup procedures.

Agriculture is one of the largest employers in Egypt [18]. The primary agricultural product in Egypt is cotton, and because of its national economic importance, the use of pesticides on that crop is highly regulated by the Egyptian Ministry of Agriculture (MOA) [19]. The national government purchases and sells the country’s entire cotton production, and once farmers agree to plant cotton, applications of chemicals on those fields come under the control of the Ministry of Agriculture. Thus, all pesticides, equipment, and calibration procedures are standardized across the Governorate*.* Adolescents are hired by the MOA as seasonal workers to apply pesticides and may work for repeated seasons.

### Intervention description

We worked with the MOA to identify feasible and appropriate methods to reduce pesticide exposure. Initial workplace observations had identified behaviors during mixing, loading, and applying pesticides that increased an adolescent’s contact with pesticides (e.g., mixing with hands, contact with pesticides during loading and applying, reentry into sprayed fields) and therefore exposure. Additionally, self-reported hygiene practices indicated variability in time to change clothes or bathe after applying pesticides. We also found that increased urinary metabolite levels were associated with increased time applying, and lower urinary metabolite levels were associated with bathing immediately after work and using a stick to mix pesticides (instead of hands) [20]. Focus groups held separately with officials from the MOA, adolescents, and parents presented study findings and discussed feasible methods to reduce exposure during application. We found that while some PPE is supplied by the MOA (masks, gloves, glasses) there is not enough for all workers, and some workers had concerns about the quality and effectiveness of the supplied PPE. Although the MOA has adopted procedures to reduce exposure (e.g., point nozzle downward, maintain distance between applicators, consider wind direction when applying), applicators reported receiving no formal training. Most workers reported bathing after work, however, there was variation in how frequently work clothes were washed (e.g., daily, monthly). The intervention incorporated feedback from the focus group in the intervention.

To decrease the burden of pesticide exposure among adolescent applicators, an educational intervention was designed and provided to them. The intervention included a training for participants about the dangers of and potential preventive measures for the use of pesticides. The training also included information about pesticides, e.g., their types, hazards they pose on the adolescents’ developing bodies, as well as the sources and modes of exposure among this group of adolescents. Three behaviors were targeted: staying out of fields recently sprayed, using a stick (instead of hands) to mix pesticides, and bathing/wearing clean clothes. The goal of the intervention was to impact the direct activities taken by the adolescents themselves. This allows the adolescent to reduce their exposure to pesticides regardless of their workplace or available resources.

Training was conducted once in each of the field offices in four districts in May 2017 before the beginning of the 2017 application season. The training was presented in a classroom format and lasted for approximately 1 h. To study the effect of the training, a pre and post intervention survey was administered on the same day. Participants were asked about their views of pesticide safety and the effectiveness of targeted behaviors to reduce exposure to pesticides before and after the intervention. A follow-up survey was administered 8 months later to assess retention. Practices of pesticide applicators were assessed before and after the training using an observational checklist administered by trained research staff. This assessment occurred in August 2016 and August 2017. Research staff were present during pesticide application throughout the multiyear study. Participants were unaware of when specific elements of the observational checklists were collected. Observational checklists included personal protective equipment worn before and during pesticide application, mixing procedures, and hygiene after pesticide application. Consent of human subjects was obtained from participants and procedures approved by the University of Iowa Institutional Review Board and the Medical Ethics Committee at Menoufia University.

All participants that attended the training (*n* = 119) completed the pre and post-training pesticide safety and behavior survey, while 95 responded to the survey 8 months after the training. Among the 119 who attended the training, 87 had completed observational checklists of pesticide application during August 2016 and 92 had observational checklists completed during August 2017. There was a total of 71 participants that completed the intervention training and had observational checklist data for 2016 and 2017.

### Risk behavior diagnosis scale

The results of the survey were used to categorize individuals using the Risk Behavior Diagnosis (RBD) Scale based on pathways from the Extended Parallel Process Model (EPPM) [21, 22]. The EPPM has been widely used in health promotion and disease prevention to develop interventions among diverse international populations [23–28].

The EPPM pathways identify how individuals are likely to control a health risk based on threat perception and efficacy of controlling that threat [22]. These pathways have been used to group individuals into four quadrants of behavior using the RBD Scale: responsive (high threat-high efficacy), pro-active (high threat-low efficacy), avoidant (low threat-high efficacy), and indifferent (low threat-low efficacy) [29, 30]. The questions used to assess threat and efficacy were related to the targeted behaviors of the interventions — mixing pesticides with a stick (not your hand), not entering fields, and hygiene around pesticide application. An average response of agree [4] on a 5-point scale of strongly disagree [1] to strongly agree [5] was used to place individuals into levels of high or low efficacy and high or low threat. Individuals were surveyed before the intervention, immediately following the intervention and 8 months post-intervention. In addition to the questions used to construct the RBD Scale, several questions about pesticide safety were asked at the same time periods.

### Internal reliability of perceived efficacy and perceived threat

The questions used to assess perceived efficacy and perceived threat are given in Table 1. Evaluation of the internal consistency of the RBD scale is given in Table 2. The internal consistency of perceived efficacy was calculated using Cronbach’s alpha and found to be 0.641. Cronbach’s alpha estimates reliability for measurement scales used in research [31]. No absolute cutoff for reliability exists, but a minimum for fair reliability of 0.60 and 0.65 has been suggested for studies of less than 100 participants and studies of 100 to 300 participants respectively [32]. Removing any individual question did not meaningfully change the internal consistency (< 1% change) of perceived efficacy and removing multiple measures only marginally increased consistency, so the final scale included all 6 questions (removal of Q11 and Q12 increase Cronbach’s alpha to 0.661). The measures of perceived threat had a Cronbach’s alpha of 0.639. Removing Q13 and Q14 improve the internal consistency and were excluded from the scale in analysis (Cronbach’s alpha = 0.690, Table 2).

**Table 1:** Risk Behavior Diagnosis Scale Questions to Classify Adolescent and Young Adult Pesticide Applicators Perceived Efficacy and Threat

Scale	Question
Perceived Efficacy	Q11 Using a stick instead of my hands to mix pesticides will prevent me from getting sick
	Q12 I am able to use a stick instead of my hands to mix pesticides
	Q15 Staying out of fields that were recently sprayed will prevent me from getting sick
	Q16 I can stay out of fields that were recently sprayed
	Q19 Changing into clean clothes after working with pesticides will prevent me from getting sick
	Q20 I can wear clean clothes with no pesticide residues
Perceived Threat	Q9 Using hands to mix pesticides can cause sickness
	Q10 If I mix pesticides with my hands it could make me sick
	Q13 Entering fields that were recently sprayed will cause sickness
	Q14 If I enter a field that was recently sprayed I am likely to get sick
	Q17 Wearing clothes with pesticide residues on them for several hours will cause sickness
	Q18 If I wear clothes with pesticide residues for several hours, I am likely to get sick

**Table 2:** Internal Consistency of the Risk Behavior Diagnosis Scale for Adolescent Applicators in Egypt

Scale	Cronbach’s alpha	Adjustment Made	Median Score (range)
Perceived Efficacy	0.641		24 (17–30)
Perceived Threat	0.639		
	0.627	Remove Q13^a^	
	0.631	Remove Q14^b^	
	0.690	Remove Q13&14	16 (10–20)

^a^Q13 Entering fields that were recently sprayed will cause sickness

^b^Q14 If I enter a field that was recently sprayed I am likely to get sick

### Statistical methods

The internal reliability of the RBD scale was assessed using Cronbach’s alpha coefficient. The scale was then used to place individuals into the four RBD quadrants of health risk behavior. Differences in demographics and pesticide application practices were compared across the quadrants using the Chi-squared test. Change in behavior post-intervention and 8 months later was compared using the McNemar test to detect a difference in the proportion of individuals placed in each RBD quadrant. Similarly, changes in patterns of PPE use found during the 2016 and 2017 observational checklist and changes in attitudes regarding pesticide safety were compared with the McNemar test. Data analysis was completed with SAS 9.4 (SAS Institute, Cary, NC) with an alpha of 0.05 used for testing significance.

## Results

### Analysis of the risk behavior diagnosis (RBD) scale

During the intervention, 119 individuals completed pre and post-surveys and participated in the educational intervention. The median age for participants was 18 years old. Participants were placed into four quadrants (Responsive, Avoidant, Proactive, or Indifferent) based on their responses to the pre-intervention survey using the RBD scale. Table 3 presents the demographic differences across RBD quadrants. More than a third of individuals (38.7%) were classified as responsive, having both a high threat and high efficacy perceptions. The second most common quadrant was indifferent (26.0%) made up of individuals with low threat and low efficacy perceptions. Across the quadrants, there were no statistically significant differences in age or pesticide application history, but those classified as indifferent were older and more likely to apply pesticides as a private applicator.

**Table 3:** Demographic Characteristics of Adolescent Applicators

Variable	Level	Total		Responsive (HT,HE)		Avoidant (HT,LE)		Proactive (LT, HE)		Indifferent (LT,LE)		P^a^
		n	%	n	%	n	%	n	%	n	%	
Age	13–17	53	44.5	21	45.7	10	58.8	13	52	9	29	
	18+	66	55.5	25	54.3	7	41.2	12	48	22	71	0.171
Applying Pesticides at MOA	No	27	22.7	10	21.7	4	23.5	4	16	9	29	
	Yes	92	77.3	36	78.3	13	76.5	21	84	22	71	0.71
Years worked for MOA	3–4	32	34.8	10	27.8	4	30.8	10	47.6	8	36.4	
	5+	60	65.2	26	72.2	9	69.2	11	52.4	14	63.6	0.49
Pesticides Private applicator	No	93	78.2	39	84.8	15	88.2	20	80	19	61.3	
	Yes	26	21.8	7	15.2	2	11.8	5	20	12	38.7	0.06
Applying Pesticides at Family fields	No	44	37	16	34.8	8	47.1	12	48	8	25.8	
	Yes	75	63	30	65.2	9	52.9	13	52	23	74.2	0.284
Hours^b^	< 3	40	33.6	15	32.6	7	41.2	6	24	12	38.7	
	> = 3	79	66.4	31	67.4	10	58.8	19	76	19	61.3	0.604

Note: *HT* High Threat, *LT* Low Threat, *HE* High Efficacy, *LE* Low Efficacy, *MOA* Ministry of Agriculture

^a^The parametric *p*-value is calculated by Chi-square test comparing levels of each variable

^b^Typical hours spent applying pesticides on days when pesticides are applied

How the intervention impacted perceived threat and efficacy is presented in Table 4 for individuals who completed surveys at all three time points (*n* = 95, 79.8%; pre-intervention, post-intervention, and 8 months after the intervention). Changing from a low efficacy or low threat category to a high category was considered an improvement. This reflects an increased awareness of the effectiveness of protective actions or the threat posed by an activity. Responsive individuals will be the most motivated to change their actions and protect themselves from a hazard. The shifts in RBD quadrant post-intervention and 8 months after the intervention were evaluated. Post-intervention, there was an increase in the proportion of individuals in the responsive quadrant (90.5%) versus the proportion of responsive individuals pre-intervention (42.1%, *p* < 0.001). The proportion of individuals in the two categories of low threat (proactive and indifferent) decreased post-intervention (*p* < 0.001). In the 8-month follow-up, most individuals were in the responsive category, but this was not significantly different from the proportion pre-intervention. There was a significant decrease in the proportion of individuals in the proactive category (*p* = 0.007) with more than half increasing their identification of threats (*n* = 11, 55.0%) and becoming responsive.

**Table 4:** Risk Behavior Diagnosis Scale Changes Over Time

Variable	Level	Pre-Intervention		Post-Intervention			8-Month Post		
		n	%	n	%	*P*-value^a^	n	%	*P*-value^a^
Responsive	No	55	57.9	9	9.5		45	47.4	
	Yes	40	42.1	86	90.5	<.001	50	52.6	0.157
Avoidant	No	81	85.3	88	92.6		72	75.8	
	Yes	14	14.7	7	7.4	0.071	23	24.2	0.083
Proactive	No	75	78.9	94	98.9		88	92.6	
	Yes	20	21.1	1	1.1	<.001	7	7.4	0.007
Indifferent	No	74	77.9	94	98.9		80	84.2	
	Yes	21	22.1	1	1.1	<.001	15	15.8	0.257

^a^The parametric p-value is calculated by McNemar test

### Intervention questionnaire responses

The responses to the additional questions surveyed before, after, and with an 8-month delay are presented in Table 5. Responses were categorized as the desired response versus a neutral response or worse (e.g., Agree vs. Disagree/Neutral or Disagree vs. Agree/Neutral where appropriate). Similar to the changes in RBD quadrant there was a strong shift in attitudes with a heightened awareness of the dangers posed by pesticide use immediately following the intervention, with a drop in this awareness when surveyed 8 months later. The proportion of respondents who viewed pesticides as posing a long-term health risk increased from 74.7 to 97.9% (*p* < 0.001) post-intervention. This change remained significant for the survey at the 8-month delay (90.5%) compared to before the intervention survey (*p* < 0.001). Several questions about hygiene had a sustained change in the proportion of individuals responding positively (Q18: wearing clothes with pesticide residue, Q24: washing hands before eating, Q25: touching pesticides and then touching face). There was a decrease in agreement that staying out of fields would prevent sickness (Q15) or mixing with bare hands (Q22) increased pesticide exposure when comparing initial opinions and opinions 8 months later.

**Table 5:** Risk Perceptions Pre, Post, and 8 Month Post-Intervention, Adolescent Pesticide Applicators in Egypt

Variable	Level	Pre		Post			8-Month Post		
		n	%	n	%	P^a^	n	%	P^a^
1. Will exposure to pesticides cause an immediate health risk?	Unlikely	29	30.5	5	5.3		24	25.3	
	Likely	66	69.5	90	94.7		64	67.4	
	IDK	0	0.0	0	0.0	<.001	7	7.4	0.141
2. Will exposure to pesticides cause a long-term health risk?	Unlikely	21	22.1	2	2.1		5	5.3	
	Likely	71	74.7	93	97.9		86	90.5	
	IDK	3	3.2	0	0.0	<.001	4	4.2	< 0.001
3. How much risk are you exposed to while using pesticides?	No Risk/Medium	88	92.6	52	54.7		44	46.3	
	Significant Risk	7	7.4	43	45.3	<.001	51	53.7	<.001
4. Direct exposure on skin to pesticides is not harmful to human health.	Disagree/Neutral	60	63.2	87	91.6		67	70.5	
	Agree	35	36.8	8	8.4	<.001	28	29.5	0.286
5. Pesticides are a greater risk to adults than adolescents.	Disagree	47	49.5	92	96.8		54	56.8	
	Agree/Neutral	48	50.5	3	3.2	<.001	41	43.2	0.300
6. A pesticide would not be put on the market if it were not safe for humans to use.	Disagree	31	32.6	39	41.1		6	6.3	
	Agree/Neutral	64	67.4	56	58.9	0.032	89	93.7	<.001
7. How confident are you that you are able to prevent yourself from being exposed to pesticides?	Not Confident/Neutral	58	61.1	19	20.0		27	28.4	
	Confident	37	38.9	76	80.0	<.001	68	71.6	<.001
8. If you need advice on how to safely handle a pesticide, how confident are you that you would be able to get advice?	Not Confident/Neutral	45	47.4	14	14.7		21	22.1	
	Confident	50	52.6	81	85.3	<.001	74	77.9	<.001
9. Using hands to mix pesticides can cause sickness.	Disagree/Neutral	22	23.2	3	3.2		18	18.9	
	Agree	73	76.8	92	96.8	<.001	77	81.1	0.479
10. If I mix pesticides with my hands it could make me sick.	Disagree/Neutral	21	22.1	2	2.1		20	21.1	
	Agree	74	77.9	93	97.9	<.001	75	78.9	0.869
11. Using a stick instead of my hands to mix pesticides will prevent me from getting sick.	Disagree/Neutral	23	24.2	10	10.5		28	29.5	
	Agree	72	75.8	85	89.5	0.003	67	70.5	0.411
12. I am able to use a stick instead of my hands to mix pesticides.	Disagree/Neutral	18	18.9	2	2.1		16	16.8	
	Agree	77	81.1	93	97.9	<.001	79	83.2	0.683
13. Entering fields that were recently sprayed will cause sickness.	Disagree/Neutral	28	29.5	5	5.3		24	25.3	
	Agree	67	70.5	90	94.7	<.001	71	74.7	0.527
14. If I enter a field that was recently sprayed I am likely to get sick.	Disagree/Neutral	28	29.5	6	6.3		28	29.5	
	Agree	67	70.5	89	93.7	<.001	67	70.5	1.000
15. Staying out of fields that were recently sprayed will prevent me from getting sick	Disagree/Neutral	18	18.9	6	6.3		32	33.7	
	Agree	77	81.1	89	93.7	0.005	63	66.3	0.023
16. I can stay out of fields that were recently sprayed.	Disagree/Neutral	20	21.1	7	7.4		30	31.6	
	Agree	75	78.9	88	92.6	0.002	65	68.4	0.096
17. Wearing clothes with pesticide residues on them for several hours will cause sickness.	Disagree/Neutral	29	30.5	0	0.0		10	10.5	
	Agree	66	69.5	95	100.0	NA	85	89.5	<.001
18. If I wear clothes with pesticide residues for several hours, I am likely to get sick.	Disagree/Neutral	27	28.4	1	1.1		7	7.4	
	Agree	68	71.6	94	98.9	<.001	88	92.6	<.001
19. Changing into clean clothes after working with pesticides will prevent me from getting sick.	Disagree/Neutral	13	13.7	1	1.1		10	10.5	
	Agree	82	86.3	94	98.9	0.001	85	89.5	0.491
20. I can wear clean clothes with no pesticide residues.	Disagree/Neutral	6	6.3	1	1.1		5	5.3	
	Agree	89	93.7	94	98.9	0.059	90	94.7	0.763
21. I can be exposed to be pesticides by breathing in pesticides when they are sprayed.	Disagree/Neutral	12	12.6	2	2.1		7	7.4	
	Agree	83	87.4	93	97.9	0.002	88	92.6	0.225
22. I can be exposed to be pesticides by mixing pesticides with bare hands.	Disagree/Neutral	9	9.5	0	0.0		19	20.0	
	Agree	86	90.5	95	100.0	NA	76	80.0	0.050
23. I can be exposed to be pesticides by eating food in the field.	Disagree/Neutral	14	14.7	3	3.2		13	13.7	
	Agree	81	85.3	92	96.8	0.002	82	86.3	0.847
24. I can be exposed to be pesticides by not washing hands before eating after applying pesticides.	Disagree/Neutral	15	15.8	2	2.1		3	3.2	
	Agree	80	84.2	93	97.9	<.001	92	96.8	0.005
25. I can be exposed to be pesticides by touching pesticides and then touching your eyes or other parts of your face.	Disagree/Neutral	22	23.2	1	1.1		6	6.3	
	Agree	73	76.8	94	98.9	<.001	89	93.7	<.001
26. I can be exposed to be pesticides by splashing pesticides on my clothes.	Disagree/Neutral	39	41.1	2	2.1		28	29.5	
	Agree	56	58.9	93	97.9	<.001	67	70.5	0.101
27. I can be exposed to be pesticides by walking in fields that were recently sprayed.	Disagree/Neutral	22	23.2	5	5.3		32	33.7	
	Agree	73	76.8	90	94.7	<.001	63	66.3	0.104
28. I can be exposed to be pesticides by storing pesticides in the field station.	Disagree/Neutral	59	62.1	19	20.0		58	61.1	
	Agree	36	37.9	76	80.0	<.001	37	38.9	0.882
29. Bathing immediately after applying pesticides will reduce my exposure to pesticides.	Disagree/Neutral	7	7.4	0	0.0		14	14.7	
	Agree	88	92.6	95	100.0	NA	81	85.3	0.090
30. Wearing clothes with pesticide residues will reduce my exposure to pesticides.	Disagree/Neutral	87	91.6	92	96.8		86	90.5	
	Agree	8	8.4	3	3.2	0.132	9	9.5	0.782
31. Getting pesticides on your skin will reduce my exposure to pesticides.	Disagree	80	84.2	94	98.9		85	89.5	
	Agree/Neutral	15	15.8	1	1.1	<.001	10	10.5	0.297

Note: *NA*, Not Applicable: McNemar test not calculated when a level has zero participants

^a^The parametric p-value is calculated by McNemar test with pre-intervention as the referent group

### PPE change

The results of observed PPE usage in 2016 and 2017 are presented in Table 6. 71 participants completed all three intervention surveys and had observational checklists completed in 2016 and 2017. From 2016 to 2017, there was a significant increase in the proportion of adolescents using a respirator or molded face mask, a hat, and goggles/glasses when mixing or applying pesticides. Fewer adolescents were wearing shoes during the mixing and application of pesticides in 2017 compared to 2016. Only one adolescent was observed wearing gloves in 2017 compared to none in 2016. When mixing was observed, hands were never used to directly mix pesticides. Instead of using hands, adolescents used a tool such as a stick to mix the pesticides (data not shown).

**Table 6:** Observed Protective Equipment Use, Adolescent Pesticide Applicators in Egypt

		2016		2017		Parametric *P*-value^a^
Variable	Level	n	%	n	%	
*Mixing*						
Goggles/glasses	Yes	10	14.1	31	43.7	<.001
Gloves	Yes	0	0.0	1	1.4	NA
Long sleeves	Yes	11	15.5	3	4.2	0.020
Pants	Yes	66	93.0	71	100.0	NA
Shoes	Yes	25	35.2	2	2.8	<.001
Mask	Yes	15	21.1	51	71.8	<.001
Spraying						
Goggles/glasses	Yes	10	14.1	33	46.5	<.001
Gloves	Yes	0	0.0	1	1.4	NA
Hat	Yes	20	28.2	67	94.4	<.001
Shoes	Yes	21	29.6	7	9.9	0.004
Mask	Yes	15	21.1	52	73.2	<.001

Note: *NA* Not Applicable: McNemar test not calculated when a level has zero participants

^a^The parametric *p*-value is calculated by McNemar test

## Discussion

Adolescent and young adult pesticide applicators’ attitudes about the health risk from the use of pesticides and their ability to control this risk were investigated. We demonstrated that a theoretically-based educational intervention can impact these attitudes. Using RBD quadrants [29, 30], we found an immediate increase in the number of individuals who would be classified as responsive or high-threat-high efficacy. These individuals are the most motivated to take the necessary precautions to reduce their exposure. The effect was not sustained except for those originally identified as proactive (low threat-high efficacy). The intervention was most effective at increasing proactive individuals’ knowledge of the threat. The RBD Scale allows for the tailoring of risk communication to an individual’s perception of the risk [22]. We demonstrate that a sustained change in individual perception of risk is possible for those who initially have a proactive view of that risk. Proactive individuals are motivated by remaining disease-free rather than the perception that the exposure will adversely impact their health [30]. For those in other quadrants of the RBD Scale, an immediate change to responsive behavior was possible for adolescents’ and young adults’ views of pesticide application risk. Responsive individuals are aware of the risk posed by pesticide use and are confident in their ability to reduce exposure. Therefore, responsive individuals are expected to be the most likely group to take the action necessary to reduce their exposure [30]. The ability to move individuals into the responsive category suggests interventions can be effective across RBD quadrants if provided after an educational component addressing threats of exposure and effectiveness of controls. This strategy of using education to move an individual to responsive behavior may have applications to other risk settings and should be evaluated further.

While not the most effective method for controlling exposure, PPE use is often recommended when working with pesticides. However, only a single individual was observed wearing gloves for spraying or mixing pesticides during the 2017 observational checklist. Additionally, fewer individuals believed they could be exposed with their bare hands while mixing pesticides when comparing the 8-month survey to the initial intervention survey. This suggests a reduced perception in the exposure potential from mixing pesticides without gloves. Despite this decrease, most applicators still believed there was a potential for exposure from this activity, but almost all adolescents observed were not wearing gloves. No adolescents were observed using their bare hands to mix pesticides directly and instead used a stick for mixing. Despite no observed use of hands, nearly a quarter of individuals did not agree that mixing pesticides with hands could cause sickness (Q9). Because mixing with a stick reduces direct contact with the pesticide, those mixing the pesticide with a stick may believe gloves were unnecessary. Alternatively, being under direct observation may have increased the practice of mixing with a stick instead of bare hands. Typical practices of the applicators may include some portion of hand mixing and would be more consistent with the risk perception of hand mixing. Exposure through the hands remains a significant route of exposure and should be the focus of future efforts to reduce pesticide exposure in similar populations [33]. Previous research with adult applicators in this region has identified dermal exposure as the primary route of exposure. Chlorpyrifos exposure was measured for Egyptian cotton field workers (*n* = 12) using 24-h urine. The dermal load was calculated by the difference in the total dose and inhalation dose via workplace air samples. Dermal exposure accounted for 94–96% of the exposure [34]. While the use of gloves was rare, we did see a general increase in use of masks, goggles, and hats. The use of PPE (e.g., long sleeves and pants, gloves, mask and goggles) was addressed in the intervention.

The greatest sustained benefit of the intervention was in attitudes towards personal hygiene practices during or after the application of pesticides. Poor personal hygiene in bathing or washing clothes can increase an individual’s pesticide exposure [7, 8]. Adolescents are also more likely to exhibit poor workplace safety habits that increase the likelihood of exposure. Based on a cross-sectional study of 87 North Carolina youth age 10–17, youths reported not practicing proper safety behavior. In the study, poor perceptions of workplace safety were related to hygiene behaviors that could increase pesticide exposure [9]. We provide new evidence that adolescent pesticide applicators are particularly susceptive to changing their perceptions regarding personal hygiene after receiving an educational intervention and that this change can be sustained over time.

### Limitations

Using the RBD scale we were able to identify shifts in risk behaviors after an educational intervention. The application of the scale is limited by the individual measures. We found attitudes around entering a field recently sprayed to be a poor measure of the perceived threat and had to remove this question from our classification of the threat of pesticide use. More precision in this question may have allowed for inclusion in the final scale and would have more accurately separated individuals into quadrants. Instead, we relied on beliefs around mixing with a stick and hygiene to classify threat beliefs. The longitudinal nature of the study resulted in some loss to follow up. We did not find any differences in RBD scale classification, hours worked applying pesticides, or age when comparing those with complete information to those only available for the first intervention. Therefore, we believe loss to follow up did not bias our results. Observational checklist results could be biased by a worker’s possible awareness of the assessment. A worker may be more vigilant with safety practices while research staff were present. This potential bias was minimized by research participants being unaware of the exact activities of the research staff and research staff being present during times when the assessment was not taking place. This bias would result in participants adhering to safe practices at a higher frequency than typically enacted. Since observational checklists were administered similarly in the two time periods, we believe our results still provide valid information about improved safety behaviors for use of most of the PPE assessed.

## Conclusions

This study used a theoretical model to develop an educational intervention to reduce occupational pesticide exposure among adolescents. The development of the intervention began with observations of work practices and surveys of adolescents to identify specific behaviors associated with exposure. The intervention incorporated the EPPM to target individual behavior change on hygiene practices and behaviors that adolescents can control. While there are other methods of controlling exposure higher on the exposure control hierarchy (elimination, substitution, engineering controls) [35], these were beyond the direct control of the adolescent applicators. Policies and workplace programs may also be effective in controlling exposure but rely on buy-in from other stakeholders. Educational interventions are low cost and can be easily tailored to the workplace. However, data presented here show the need for regular training – since some results did not last into the 8-month period. This intervention, administered regularly, is a low-cost solution that can be applied in other low and middle-income countries to reduce adolescent pesticide exposure.

## Data Availability

The datasets analyzed during the current study are available from the corresponding author on reasonable request. To gain access, data requestors will need to sign a data access agreement.

## Abbreviations

ADHD: Attention-Deficit/Hyperactivity Disorder
EPPM: Extended Parallel Process Model
MOA: Ministry of Agriculture
PPE: Personal protective equipment
RBD: Risk Behavior Diagnosis
